# Spontaneous collapse as a prognostic marker for human blastocysts: a systematic review and meta-analysis

**DOI:** 10.1093/humrep/dead166

**Published:** 2023-08-15

**Authors:** Kate Bickendorf, Fang Qi, Kelli Peirce, Jay Natalwala, Vincent Chapple, Yanhe Liu

**Affiliations:** Fertility North, Joondalup, Western Australia, Australia; Systematic Review Solutions Ltd, Shanghai, China; Fertility North, Joondalup, Western Australia, Australia; Fertility North, Joondalup, Western Australia, Australia; Fertility North, Joondalup, Western Australia, Australia; Fertility North, Joondalup, Western Australia, Australia; School of Medical and Health Sciences, Edith Cowan University, Joondalup, Western Australia, Australia; School of Human Sciences, University of Western Australia, Crawley, Western Australia, Australia; School of Health Sciences and Medicine, Bond University, Robina, Queensland, Australia

**Keywords:** blastocyst, collapse, contract, meta-analysis, time-lapse, live birth, miscarriage, ploidy, expansion, shrinkage

## Abstract

**STUDY QUESTION:**

Is spontaneous collapse (SC) by human blastocysts a prognostic factor in IVF treatment?

**SUMMARY ANSWER:**

SC in human blastocyst is associated with reduced euploid embryo and pregnancy rates.

**WHAT IS KNOWN ALREADY:**

SC of the human blastocyst is a phenomenon that was revealed relatively recently following the clinical application of time-lapse monitoring in IVF laboratories. The ploidy and clinical prognosis of affected blastocysts are still poorly understood, with inconsistent reports. Systematic reviews and meta-analyses on this topic are currently absent in the literature but its potential as a marker of embryo viability holds great clinical value. In this study, we aimed to comprehensively evaluate the potential of SC as a prognostic factor in regard to ploidy status, and pregnancy, live birth and miscarriage rates.

**STUDY DESIGN, SIZE, DURATION:**

A systematic review and meta-analysis were performed according to PRISMA guidelines, with a protocol registered with PROSPERO (CRD42022373749). A search of MEDLINE, EMBASE, and the Cochrane Library for relevant studies was carried out on 10 October 2022, using key words relevant to ‘blastocyst collapse’ and ‘time-lapse imaging’.

**PARTICIPANTS/MATERIALS, SETTING, METHODS:**

Two independent reviewers systematically screened and evaluated each study in terms of participants, exposure, comparator, and outcomes (PECO). The Quality In Prognosis Studies tool was used for quality assessment. Data were extracted according to Cochrane methods. Pregnancy, live birth, ploidy, or miscarriage data were summarized by risk ratios (RRs) or odds ratios and their 95% CIs. All meta-analyses were performed with random-effects models.

**MAIN RESULTS AND THE ROLE OF CHANCE:**

Following removal of duplicates, a total of 196 records were identified by the initial search. After screening according to PECO, 19 articles were included for further eligibility assessment. For meta-analysis, seven retrospective cohort studies were eventually included. After data pooling, the incidence of blastocyst SC was 37.0% (2516/6801) among seven studies (ranging from 17.4% to 56.2%). SC was associated with significantly lower clinical pregnancy rates (two studies, n = 736; RR = 0.77, 95% CI = 0.62–0.95; *I*^2^ = 30%), ongoing pregnancy rates (five studies, n = 2503; RR = 0.66, 95% CI = 0.53–0.83; *I*^2^ = 60%), and reduced euploidy rates (three studies, n = 3569; RR = 0.70, 95% CI = 0.59–0.83; *I*^2^ = 69%). Nevertheless, live birth rates (two studies, n = 816; RR = 0.76, 95% CI = 0.55–1.04; *I*^2^ = 56%) and miscarriage rate (four studies, n = 1358; RR = 1.31, 95% CI = 0.95–1.80; *I*^2^ = 0%) did not differ between blastocysts with or without SC. There was, however, significant heterogeneity between the studies included for evaluation of ongoing pregnancy rates (*I*^2^ = 60%, *P *=* *0.04), live birth rates (*I*^2^ = 56%, *P *=* *0.13), and ploidy rates (*I*^2^ = 69%, *P *=* *0.04). Subgroup analyses were conducted according to different definitions of SC, number of collapse events, and whether the transferred blastocyst had undergone preimplantation genetic testing for aneuploidy; with inconclusive findings across subgroups.

**LIMITATIONS, REASONS FOR CAUTION:**

All studies in the meta-analysis were retrospective with varying levels of heterogeneity for different outcomes. Not all studies had accounted for potential confounding factors, therefore only unadjusted data could be used in the main meta-analysis. Studies employed slightly different strategies when defining blastocyst SC. Standardization in the definition for SC is needed to improve comparability between future studies.

**WIDER IMPLICATIONS OF THE FINDINGS:**

Our results indicate that blastocyst SC has negative implications for a pregnancy. Such blastocysts should be given a low ranking when selecting from a cohort for intrauterine transfer. Blastocyst SC should be considered as a contributing variable when building blastocyst algorithms to predict pregnancy or live birth.

**STUDY FUNDING/COMPETING INTEREST(S):**

There is no external funding to report. All authors report no conflict of interest.

**REGISTRATION NUMBER:**

PROSPERO 2022 CRD42022373749

## Introduction

ART has been clinically practiced worldwide over the past 40 years (Edwards and Steptoe, 1983). Successful treatment via IVF with the least number of cycles depends on the selection of the most viable embryo(s) from the cohort created. Extended culture to the blastocyst stage is an effective approach that has been used in most ART laboratories (Gardner and Schoolcraft, 1999). The original Gardner grading system was a three-point scoring method including the degree of blastocoel expansion, and a grading of the inner cell mass and trophectoderm (Gardner and Schoolcraft, 1999). Numerical ranking systems based on this system and employing more complex mathematical models have also been recently explored, aiming to optimize the accuracy of selection using calculated weighting for each contributing factor (Liu *et al.*, 2022). Time-lapse microscopy (TLM) on human embryos is being adopted by an increasing number of IVF clinics globally (Meseguer *et al.*, 2011; Liu *et al.*, 2020b). TLM enables observation of human embryos at a high level of detail, without the need to interrupt culture conditions, offering unique opportunities for embryologists to identify novel markers of embryo viability (Liu *et al.*, 2015; Liu *et al.*, 2016). These include qualitative time-lapse markers, such as direct cleavage and reverse cleavage, which would go undetected using conventional static microscopic observations (Rubio *et al.*, 2012; Liu *et al.*, 2014b). These markers differ from the morphokinetic parameters which were shown to be prone to inter-operator inconsistencies, and they are thought to be less affected by inter-laboratory variations in the embryo culture conditions (Liu *et al.*, 2020a). Future artificial intelligence (AI) tools may hold the potential to further improve the annotation process for such markers from both the workflow and consistency perspectives.

Blastocyst spontaneous collapse (SC) is one of a number of recently reported potential qualitative time-lapse markers of embryo viability (Marcos *et al.*, 2015). SC, alternatively known as shrinkage or contraction, can occur in human blastocysts during the expansion process up to Day 7, usually followed by re-expansion. Considering the degree of shrinkage and time spent re-expanding, different definitions for SC were reported (Bodri *et al.*, 2016; Gazzo *et al.*, 2020; Cimadomo *et al.*, 2022). However, clinical evidence in the literature regarding the long-term prognosis following transfer of such SC blastocysts is limited, sometimes with inconsistent conclusions (Marcos *et al.*, 2015; Bodri *et al.*, 2016). Emerging studies have suggested that episodes of blastocyst SC may also be used for blastocyst de-prioritization (Cimadomo *et al.*, 2022). However, its role in the subsequent hatching process and the biological mechanism behind it remain unclear (Sciorio and Meseguer, 2021). Furthermore, a systematic review followed by meta-analysis with synthesized evidence is still absent in literature. Therefore, in this study, we aimed to comprehensively evaluate the potential of SC as a prognostic factor with regard to ploidy status, pregnancy rate, live birth rate, and miscarriage rate.

## Materials and methods

We performed this systematic review and meta-analysis in accordance with the standards of methodological logic described in the Cochrane Handbook (Higgins *et al.*, 2022) and reported in accordance with the Preferred Reporting Items for Systematic Reviews and Meta-Analyses (PRISMA) criteria (Page *et al.*, 2021). The protocol was registered with PROSPERO (CRD42022373749).

### Search strategies

We searched PubMed, Embase, and the Cochrane Library on 10 October 2022 using the keywords ‘blastocyst collapse’ (‘spontaneous collapse’ or ‘blastocyst contractions’ or ‘blastocyst shrinkage’ or ‘blastocyst expansion’ or ‘embryo kinetics’ or ‘embryonic competence’) and ‘Time-Lapse Imaging’ (‘Time Lapse’ or ‘Time Lapsed’), with no restrictions on date, language, document type, or publication status. The detailed search strategy for each database is presented in the Supplementary Materials and Methods.

### Eligibility criteria and study selection

All controlled studies, either prospective or retrospective, including randomized controlled trials and non-randomized controlled studies (non-randomized trials and observational study design of cohort, case-control types) fulfilling the following criteria were included: women, regardless of age or number of previous attempts, who were treated with IVF or ICSI and whose resulting embryos were observed using TLM; the comparison was between blastocysts displaying SC (single or multiple, definition used by each of the included studies) during culture and those not showing collapse; data available on any one outcome of interest, including the primary outcomes pregnancy rate (where a clinical pregnancy is defined as clinical evidence of gestational sac or foetal heart beat under ultrasound, and ongoing pregnancy defined as stated in each included study) and live birth rate, and secondary outcomes euploidy rate and miscarriage rate, as defined in each included study.

Two reviewers (K.B. and F.Q.) screened the search results independently. Once duplicate records had been removed and all remaining references initially screened by title and abstract, full-text reports deemed to meet the eligibility criteria were obtained for further review followed by a final decision on inclusion. Any disagreements were resolved by discussion between the two reviewers, assisted by a third reviewer (Y.L.) if necessary.

### Data extraction and quality assessment

Two reviewers (K.B. and F.Q.) independently extracted quantitative and qualitative information on participants, comparisons, and outcomes, using a standardized data collection Excel (Microsoft^®^ Excel^®^, Microsoft Corporation, Redmond, WA, USA) form. Any disagreements were resolved by discussion between the two reviewers, assisted by a third reviewer (Y.L.) if necessary. We used the Quality In Prognosis Studies tool to assess the quality of non-randomized controlled studies, including study participation, study attrition, prognostic factor measurement, outcome measurement, study confounding, statistical analysis, and reporting (Hayden *et al.*, 2013).

### Data analysis

All dichotomous outcomes were summarized by risk ratios (RRs) and their 95% CIs. We conducted all meta-analyses with random-effects models using RevMan 5.4 software. Subgroup analysis was performed where possible according to whether or not blastocysts had undergone preimplantation genetic testing for aneuploidy (PGT-A). In addition, we also synthesized data for the primary outcomes using fixed-effect models to test the robustness of results. We fully discussed the clinical and methodological heterogeneity to ensure the homogeneity of included studies before meta-analysis. An *I*^2^ > 50% coupled with the significance of *χ*^2^ test (*P *<* *0.1) was considered substantial level of statistical heterogeneity, with possible sources of the heterogeneity investigated. Subgroup analyses according to female age or insemination method were not performed as planned owing to insufficient data.

## Results

### Study selection

**Figure 1.**
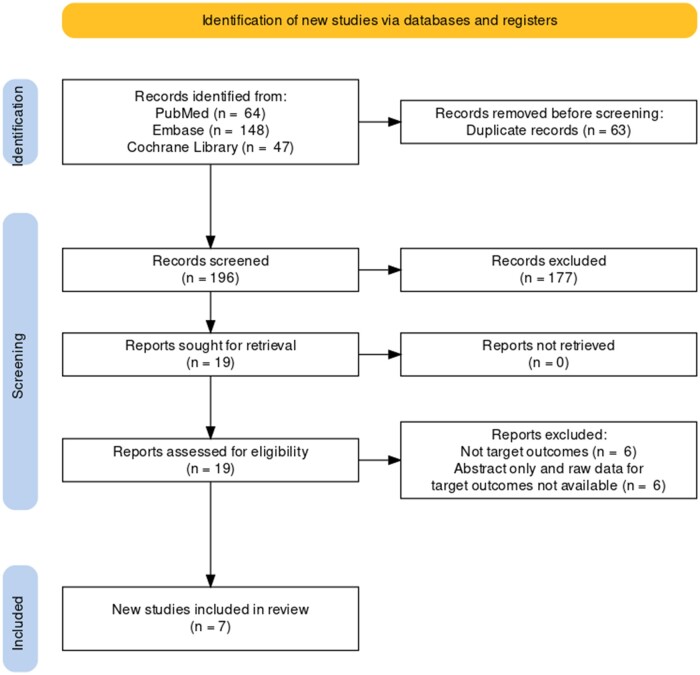
**PRISMA flow diagram of the identification of studies for a systematic review of human blastocyst spontaneous collapse.** PRISMA: Preferred Reporting Items for Systematic Reviews and Meta-Analyses.

The initial search resulted in 259 records through all databases, and after removal of duplicates 196 unique records remained to be screened. Following inspection of titles and abstracts, we excluded 177 records. The remaining 19 records were read in full, with 12 records subsequently excluded for different reasons, as specified in Fig. 1. For example, Esbert *et al.* (2017) exclusively focused on the contribution of assisted hatching to the frequency of SC, and Gilboa *et al.* (2021) evaluated weak contractions or ‘pumping’ instead of significant SC. Finally, seven studies (Marcos *et al.*, 2015; Bodri *et al.*, 2016; Viñals Gonzalez *et al.*, 2018; Gazzo *et al.*, 2020; Sciorio *et al.*, 2020a,b; Cimadomo *et al.*, 2022) were included in this systematic review and meta-analysis.

### Study characteristics and quality assessment

All included studies were retrospective cohort studies with women from Italy, Japan, Mexico, Spain, the UK, and the USA. Women’s mean age ranged from 30.4 (Gazzo *et al.*, 2020) to 38.8 years (Cimadomo *et al.*, 2022). Sample size varied from 277 (Bodri *et al.*, 2016) to 2348 (Cimadomo *et al.*, 2022). The definition of blastocyst SC, as where ≥50% of the trophectoderm surface was separated from the zona pellucida, was consistent amongst five studies (Marcos *et al.*, 2015; Bodri *et al.*, 2016; Viñals Gonzalez *et al.*, 2018; Sciorio *et al.*, 2020a,b). However, different cut-off values were used in the other two studies (Gazzo *et al.*, 2020; Cimadomo *et al.*, 2022) (Table 1). After data pooling, the incidence of blastocyst SC was 37.0% among the seven included studies, ranging from 17.4% (Sciorio *et al.*, 2020b) to 56.2% (Viñals Gonzalez *et al.*, 2018). Assisted hatching was used in two studies (Viñals Gonzalez *et al.*, 2018; Gazzo *et al.*, 2020), while one other study involved vitrified-warmed oocytes (Marcos *et al.*, 2015). No additional intervention was reported in the other three studies (Bodri *et al.*, 2016; Sciorio *et al.*, 2020a,b). Study characteristics are detailed in Table 1.

**Table 1.** Characteristics of studies included in a systematic review of human blastocyst spontaneous collapse.

Study ID	Country	Mean age of population (years)	Insemination method	Total number of embryos included	Incidence of collapse	Definition of collapse	Additional interventions	Outcomes reported
Bodri *et al.* (2016)	Japan	38.4	IVF or ICSI	277	45.8% (127/277)	>50% of the surface of the TE was separated from the ZP	None	Live birth rate per SET
Cimadomo *et al.* (2022)	Italy	38.8	ICSI	2348	49.7% (1168/2348)	A continuous reduction of the ZP area occurs after tSB, the process lasts <10 h and the embryo: ZP ratio (area of the collapsed embryo/area of the ZP) at the end of the process is ≤90%.	PGT-A	Euploidy; Live birth rate per vitrified-warmed euploid SET; Miscarriage among euploid blastocysts
Gazzo *et al.* (2020)	Mexico USA	30.4	IVF	912	28.2% (257/912)	≥20% of the surface of the TE was separated from the ZP.	Day 4 assisted hatching and PGT-A	Euploidy; Ongoing pregnancy among euploid blastocysts
Marcos *et al.* (2015)	Spain	38.1	IVF or ICSI	715	19.4% (139/715)	≥50% of the surface of the TE was separated from the ZP.	Oocyte vitrification and warming	Clinical pregnancy; Ongoing pregnancy; Miscarriage
Sciorio *et al.* (2020a)	Spain UK	NR	IVF or ICSI	1297	19.9% (259/1297)	A blastocyst volume reduction ≥50%	None	Ongoing pregnancy;
Sciorio *et al.* (2020b)	UK	32.7	IVF or ICSI	356	17.4% (62/356)	A blastocyst volume reduction ≥50%	None	Ongoing pregnancy; Miscarriage
Viñals Gonzalez *et al.* (2018)	UK	38.1	ICSI or IMSI	896	56.2% (504/896)	≥50% of the surface of the TE was separated from the ZP	Day 3 assisted hatching and PGT-A	Euploidy; Clinical and ongoing pregnancy among euploid blastocysts; Miscarriage among euploid blastocysts

IMSI: intracytoplasmic morphologically selected sperm injection; NR: not reported; PGT-A: preimplantation genetic testing for aneuploidy; SET: single embryo transfer; TE: trophectoderm; tSB: time of initiation of blastulation; ZP: zona pellucida.

The risk of bias for each study was moderate overall (Supplementary Table S1). Owing to the retrospective nature of all included studies, confounders may not always be adjusted for when analysing the outcomes of interest, as listed earlier.

### Meta-analyses for all outcomes

#### Pregnancy rate

**Figure 2.**
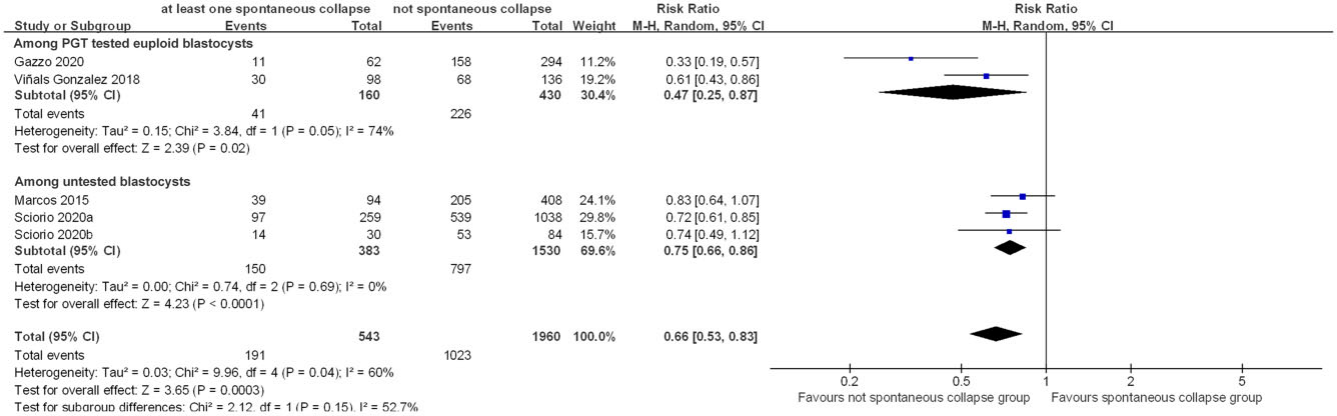
**Association of spontaneous collapse of human blastocysts with ongoing pregnancy rates.** Subgroup analyses for blastocysts that underwent preimplantation genetic testing (PGT) for aneuploidy or untested blastocysts were performed.

**Figure 3.**
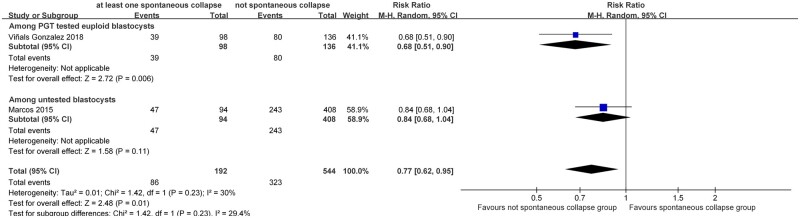
**Association of clinical pregnancy rates with spontaneous collapse of human blastocysts.** Subgroup analyses for blastocysts that underwent preimplantation genetic testing (PGT) for aneuploidy or untested blastocysts were performed.

Five included studies (Marcos *et al.*, 2015; Viñals Gonzalez *et al.*, 2018; Gazzo *et al.*, 2020; Sciorio *et al.*, 2020a,b) reported ongoing pregnancy rates, of which two studies (Marcos *et al.*, 2015; Viñals Gonzalez *et al.*, 2018) also reported clinical pregnancy rates. Results indicated that transfer of blastocysts with at least one SC led to significantly lower ongoing pregnancy rates than embryos without SC (five studies, n = 2503; RR = 0.66 (0.53, 0.83)_95% CI_; *I*^2^ = 60%; Fig. 2). Considering the substantial heterogeneity identified, we further explored potential contributors by conducting subgroup analysis based on whether the blastocyst had undergone PGT-A. Subgroup analysis showed that both euploid (two studies, n = 590 RR = 0.47 (0.25, 0.87)_95% CI_; *I*^2^ = 74%) and untested blastocysts (three studies, n = 1913; RR = 0.75 (0.66, 0.86)_95% CI_; *I*^2^ = 0%) had reduced ongoing pregnancy rates when displaying at least one SC in reference to those without (Fig. 2). Similarly, clinical pregnancy rate was also lower for SC blastocysts than their non-collapsing counterparts (two studies, n = 736; RR = 0.77 (0.62, 0.95)_95% CI_; *I*^2^ = 30%; Fig. 3). The level of heterogeneity for clinical pregnancy rate analysis was considered acceptable, with subgroup analysis implying a similar trend among euploid blastocysts (one study, n = 234; RR = 0.68 (0.51, 0.90)_95% CI_) and untested blastocysts (one study, n = 502; RR = 0.84 (0.68, 1.04)_95% CI_) (Fig. 3). Following sensitivity analyses (random-effects model versus fixed-effect model), the conclusions appeared to be consistent (Supplementary Figs S1 and S2).

Within the five included studies, only Sciorio *et al.* (2020a) reported results using a multiple regression model, indicating a significant effect of SC on ongoing pregnancy rates (Table 2).

**Table 2.** Results from a multiple regression model assessing associations between the proportion of human blastocysts with spontaneous collapse and outcomes.

Outcome	Study ID	Comparisons	OR	95% CI	*P*	Adjusted factors
Ongoing pregnancy rates	Sciorio (2020a)	At least one blastocyst collapse versus no blastocyst collapse	0.5	0.36 to 0.7	<0.001	Type of cycle; age; blastocyst morphology; clinic centre; fertilization method
Live birth rates	Bodri (2016)	Single blastocyst collapse versus no blastocyst collapse	1.87	0.8 to 4.4	0.148	Eight TLM variables and eight patient related and cycle-related confounders
	Bodri (2016)	Multiple blastocyst collapse versus no blastocyst collapse	0.86	0.26 to 2.74	0.803	Eight TLM variables and eight patient related and cycle-related confounders
Cumulative live birth rates	Cimadomo (2022)	Rate of collapsing embryos (either viable or subsequently degenerating) per cycle	0.91	0.55 to 1.5	0.7	Maternal age and number of MII oocytes
Euploid embryo rates	Cimadomo (2022)	At least one blastocyst collapse versus no blastocyst collapse	0.78	0.62 to 0.98	0.03	Oocyte age; blastocyst quality; t-biopsy; blastocyst area at t-biopsy

MII: metaphase II; OR: odds ratio; t: time; TLM: time-lapse microscopy.

#### Live birth rate

**Figure 4.**
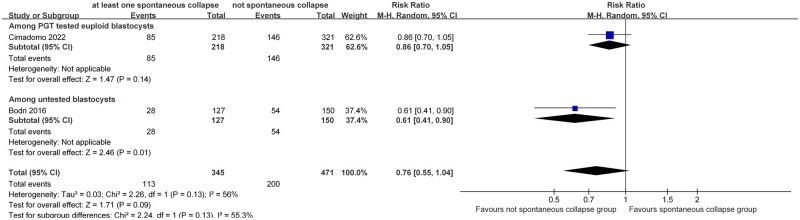
**Association of spontaneous collapse of human blastocysts with live birth rates.** Subgroup analyses for blastocysts that underwent preimplantation genetic testing (PGT) for aneuploidy or untested blastocysts were performed.

Two included studies (Bodri *et al.*, 2016; Cimadomo *et al.*, 2022) reported live birth rates. Blastocysts with at least one SC resulted in lower live birth rates than those showing no SC (two studies, n = 816; RR = 0.76 (0.55, 1.04)_95% CI_; *I*^2^ = 56%; Fig. 4). Following sensitivity analyses, narrower CIs were obtained by using fixed-effects model (two studies, n = 816; RR = 0.78 (0.65, 0.94)_95% CI_; *I*^2^ = 56%; Supplementary Fig. S3). Considering the substantial heterogeneity identified, we further explored potential contributors and conducted subgroup analysis according to the use of PGT-A. Euploid blastocysts with at least one SC led to lower live birth rate than those without (Cimadomo *et al.*, 2022), although the difference was not statistically significant (one study, n = 539; RR = 0.86 (0.70, 1.05)_95% CI_; Fig. 4). However, untested blastocysts with at least one SC resulted in a significantly reduced live birth rate than those without (Bodri *et al.*, 2016) (one study, n = 277; RR = 0.61 (0.41, 0.90)_95% CI_; Fig. 4).

Both studies (Bodri *et al.*, 2016; Cimadomo *et al.*, 2022) also separated single and multiple SCs versus no SC. Meta-analysis did not detect a significant difference in live birth rates comparing blastocysts with single SC to those without SC (two studies, n = 677; RR = 0.94 (0.77, 1.14)_95% CI_; *I*^2^ = 0%; Supplementary Fig. S4); but did detect a significantly decreased live birth rate comparing blastocysts with multiple SCs to those without (two studies, n = 610; RR = 0.53 (0.31, 0.92)_95% CI_; *I*^2^ = 55%; Supplementary Fig. S5) Following multiple regression analysis, Bodri *et al.* (2016) reported no clear associations between number of blastocyst SC events and live birth after adjusting potential confounding factors (Table 2). In addition, Cimadomo *et al.* (2022) reported no association between the proportion of SC blastocysts (either viable or subsequently degenerating) and cumulative live birth rates per egg collection (Table 2).

#### Euploidy rates

**Figure 5.**
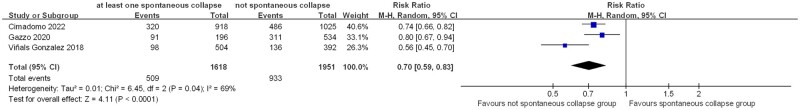
**Association of euploidy rate with spontaneous collapse of human blastocysts**.

Three included studies (Viñals Gonzalez *et al.*, 2018; Gazzo *et al.*, 2020; Cimadomo *et al.*, 2022) reported euploidy rates. Meta-analysis indicated that blastocysts with at least one SC had lower euploidy rates than those without (three studies, n = 3569; RR = 0.70 (0.59, 0.83)_95% CI_; *I*^2^ = 69%; Fig. 5). All included studies indicated the same trend, although no clear sources of the heterogeneity were identified.

Cimadomo *et al.* (2022) also separately assessed blastocysts with single and multiple SCs in reference to those without, where both single (one study, n = 1565; RR = 0.81 (0.71, 0.92)_95% CI_) and multiple SCs (one study, n = 1403; RR = 0.63 (0.53, 0.75)_95% CI_) were associated with lower euploidy rates (Supplementary Figs S6 and S7). Using a multiple regression model, the same group also established a correlation between SC occasions and euploidy (Table 2).

#### Miscarriage rate

**Figure 6.**
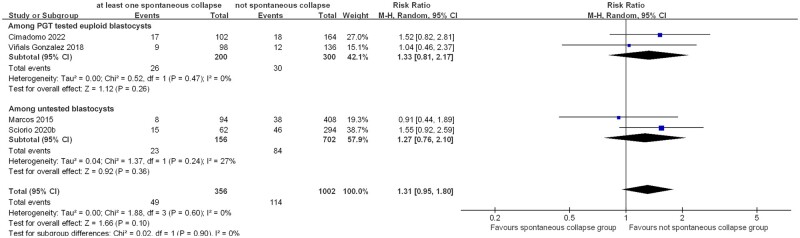
**Association of spontaneous collapse of human blastocysts with miscarriage rate.** Subgroup analyses for blastocysts that underwent preimplantation genetic testing (PGT) for aneuploidy or untested blastocysts were performed.

Four included studies (Marcos *et al.*, 2015; Viñals Gonzalez *et al.*, 2018; Sciorio *et al.*, 2020b; Cimadomo *et al.*, 2022) reported miscarriage rates. Pooled analysis indicated that transfer of blastocysts with at least one SC resulted in higher miscarriage rates than those without, albeit not statistically significant (four studies, n = 1358; RR = 1.31 (0.95, 1.80)_95% CI_; *I*^2^ = 0%; Fig. 6). The comparisons remained statistically insignificant after separating euploid blastocysts (two studies, n = 500; RR = 1.33 (0.81, 2.17)_95% CI_; *I*^2^ = 0%) and untested blastocysts (two studies, n = 858; RR = 1.27 (0.76, 2.10)_95% CI_; *I*^2^ = 27%) (Fig. 6).

## Discussion

In this systematic review and meta-analysis, we investigated the prognostic outcomes of blastocysts showing SC and managed to include seven retrospective cohort studies. Outcomes of interest included ploidy status, pregnancy rate, live birth rate, and miscarriage rates. However, not every included study had accounted for potential confounding factors for these outcomes, therefore we could only use unadjusted data in the main meta-analysis. While the seven studies included a diverse population with heterogeneous clinical settings, SC definitions, annotation strategies, and geographic locations (Table 1), there were no clear definitions of what determines an ongoing pregnancy or miscarriage; therefore, it was assumed that each group of authors based their definitions on consensus. It should also be noted that assisted hatching performed in two of the included studies (Viñals Gonzalez *et al.*, 2018; Gazzo *et al.*, 2020) may present as a source of variability in our analysis. Further, the reviewing process in this meta-analysis was not free from bias, involving subjective decisions including selection criteria, data extraction, and statistical analysis. Nevertheless, with pooled data from seven studies, we highlighted a 37.0% (n = 6801) incidence rate of blastocyst SC, ranging from 17.2% to 56.2% as reported by different groups. The true incidence rate of SC could be higher because of the limited observation time of blastocysts before the *in vitro* culture ended for intrauterine transfer and cryopreservation (Liu *et al.*, 2022). Substantial evidence was identified by our meta-analysis reinforcing the adverse impact of blastocyst SC on the subsequent prognosis, including reduced rates of euploidy, clinical pregnancy, and ongoing pregnancy; the differences in live birth and miscarriage rates were, however, not statistically significant. Despite the retrospective nature of all seven studies, our synthesized data do hold clinical value to guide daily practice, pinpointing the value of blastocyst SC as a potential viability marker for blastocyst deselection.

It is important to highlight that significant heterogeneity was detected amongst studies while analysing most outcome endpoints. Subgroup evaluation was conducted to combat this, but certain comparisons subsequently lost statistical significance with the reduced sample size. Apart from the different blastocyst populations (i.e. untested versus euploid), which was used as a subgrouping criterion, other sources of heterogeneity could not be included for subgrouping because the data were unavailable. For example, firstly, patient cohorts were radically different between the Marcos *et al.* (2015) and Bodri *et al.* (2016) studies, with more than half of the patient population in the former study being young oocyte donors, whereas all patients involved in the latter study were infertile with more advanced age. Secondly, samples sizes varied significantly. Bodri *et al.* (2016) recorded 277 single blastocyst transfers, while Cimadomo *et al.* (2022) recorded 2348. Thirdly, Marcos *et al.* (2015), Sciorio *et al.* (2020a), and Sciorio *et al.* (2020b) included fresh transfers only, while others included blastocysts that had been previously vitrified and subsequently warmed. This may potentially involve embryo-endometrial asynchrony as an extra confounder, especially when Day 6 blastocysts were transferred in a frozen cycle (Tannus *et al.*, 2019). Furthermore, the definition of SC was not consistent. Most studies defined SC as a volume reduction of ≥50%, while Cimadomo *et al.* (2022) outlined that there had to be an uninterrupted reduction of the zona pellucida lasting for 10 h or less and the final embryo:zona pellucida ratio equivalent to or <90%. In addition, Gazzo *et al.* (2020) defined a collapse episode as a reduction in size of at least 20%. It is therefore acknowledged that such inconsistent definitions of SC may impact data interpretation when assessing its impact on the treatment outcomes. Future studies employing a standardized definition of SC with more robust measurement tools, such as the one used in the Cimadomo *et al.* (2022) study, are required to generate higher quality evidence. It is also important to note the inevitable variation in the duration of culture time between blastocysts. However, unfortunately, such timing data was unavailable from the included studies to enable synthesized meta-analysis.

The blastocyst stage begins at 4–5 days post-fertilization in humans (Biggers *et al.*, 1977). This is when the sodium pumps in the future trophectoderm cells start to pump sodium into the centre of the embryo, so that the accumulating sodium osmotically draws in water to create a blastocoel cavity (Biggers *et al.*, 1977; Le Verge-Serandour and Turlier, 2021; Le Verge-Serandour and Turlier, 2022). The growing blastocoel forces the zona pellucida to thin, and the hatching process follows, which is mandatory for implantation to occur (Cohen *et al.*, 1985; Gonzales *et al.*, 1996). To date, the molecular and metabolic mechanisms behind blastocyst SC remain unclear. Togashi *et al.* (2015) proposed that a strong episode of blastocyst collapse could be caused by compromised trophectoderm cells, either by failing to avoid water loss by maintenance of the functionality of sodium pump or the integrity of the cell junction structure. As a significant amount of energy is required for the re-expansion process to occur (Biggers *et al.*, 1977), SC may be associated with harm to the blastocyst (Pike, 1981). This is also supported by other animal models (Massip *et al.*, 1982; Niimura, 2003), although Niimura (2003) believed a weak contraction (defined as <20% volume reduction) may benefit the hatching process. Considering this, it would be interesting to compare SC incidence in human blastocysts cultured in different media, considering the varying energy ingredients (Ciray *et al.*, 2012). Clinical studies are also needed to further explore the potential beneficial role of weak SC in blastulation and subsequent implantation, considering the plastic nature of human embryos (Coticchio *et al.*, 2021). Longer-term follow-up studies after transfer of SC blastocysts are necessary to aid in understanding its contribution to the subsequent placental complications, such as pre-eclampsia and intrauterine growth restriction, considering the potential association with a suboptimal integrity of the trophectoderm epithelium.

Clinical evidence has uncovered the poor morphology of SC blastocysts when a large shrinkage, longer recovery time and repeated occurrences are involved (Cimadomo *et al.*, 2022). Cimadomo *et al.* (2022) further reported the increased subsequent degeneration rate in blastocysts showing SC compared to their non-collapsing counterparts (21% versus 13%, respectively). Cycle-based investigation, however, suggested SC was more an expression of the intrinsic fitness of individual blastocysts rather than relating to any patient factor (Cimadomo *et al.*, 2022). Therefore, caution ought to be taken when interpreting direct comparisons in clinical outcomes between blastocysts with or without SC, because those with SC are often deprioritized for transfer and this carries intrinsic selection bias as these blastocysts are more likely to have poorer morphology, longer culture duration and originate from patients with more failed transfers. Further research focusing on patient or cycle characteristics is required to confirm this. Based on an aneuploidy dataset, Viñals Gonzalez *et al.* (2018) postulated that blastocyst SC may correlate with aneuploidies with an extra chromosome 1, 6, or 19, which carry blastulation-related genes such as Na/K-ATPase pumps and adherens/gap/tight junctions. Our synthesized data also demonstrated elevated adverse impact of multiple SCs on both euploidy rate and live birth rate. Therefore, establishing whether or not SC is purely caused by chromosome-associated changes requires more evidence. Nevertheless, the number of SC events could be used as an additional predictor of blastocyst prognosis, pending further confirmation by well-designed studies with standardized methodology.

Indeed, in addition to different definitions of SC, blastocyst assessment in general is well accepted to have intra- and inter-operator inconsistency (Sunde, 2007; Storr *et al.*, 2017). Future studies on blastocyst SC with the assistance of AI tools may offer better quality evidence supported by improved robustness in methodology. Computerized measuring tools are now already being increasingly used on human oocytes and embryos, with demonstrable improved accuracy (Ciray *et al.*, 2014; Liu *et al.*, 2014a; Orevich *et al.*, 2022). Marcos *et al.* (2015) utilized the Embryoviewer drawing tools to measure blastocyst collapse calculating whether ≥50% of the surface of the trophectoderm had separated from the zona pellucida. More recently, Cimadomo *et al.*, (2022) employed an AI algorithm for their analysis of blastocyst SC, supposedly with further improved robustness in annotation and reproducibility in measurement. Such methodology would minimize bias arising from human factors, yielding a higher quality dataset at greater speed. Compared to embryo-selection algorithms that are based on black-box training using purely raw images, such an approach combining automated embryo annotation and interpretable (biologically meaningful) parameters may help to address certain social and societal concerns regarding black-box AI, as recently underlined by Afnan *et al.* (2021).

## Conclusion

This meta-analysis highlighted the link between blastocyst SC and the subsequent poor prognostic outcomes seen in ploidy rates and pregnancy rates. Evidence suggests the potential of SC as a marker of blastocyst viability, although further studies based on a standardized definition of SC, computer assisted robust measuring methodology and increased sample size are required to draw solid conclusions on this topic.

## Supplementary Material

dead166_Supplementary_Figure_S1

dead166_Supplementary_Figure_S2

dead166_Supplementary_Figure_S3

dead166_Supplementary_Figure_S4

dead166_Supplementary_Figure_S5

dead166_Supplementary_Figure_S6

dead166_Supplementary_Figure_S7

dead166_Supplementary_Table_S1

dead166_Supplementary_Data_File

## Data Availability

All data are incorporated into the article and its online supplementary material.
