# Supplementary material for: Spontaneous collapse as a prognostic marker for human blastocysts: a systematic review and meta-analysis
Source: Hum Reprod. 2023 Aug 15;38(10):1891–900. doi: 10.1093/humrep/dead166 (PMC10546075; doi:10.1093/humrep/dead166)
Supplement: dead166_Supplementary_Figure_S1 [file dead166_supplementary_figure_s1.pdf]

OXFORD
human reproduction
Human Reproduction, 2023, 1–1
https://doi.org/10.1093/humrep/dead166
Supplementary Data

Study or Subgroup	at least one spontaneous collapse Events	Total	not spontaneous collapse Events	Total	Weight	Risk Ratio M-H, Fixed, 95% CI
**Among PGT tested euploid blastocysts**						
Gazzo 2020	11	62	158	294	12.7%	0.33 [0.19, 0.57]
Viñals Gonzalez 2018	30	98	68	136	13.2%	0.61 [0.43, 0.86]
**Subtotal (95% CI)**		**160**		**430**	**25.9%**	**0.47 [0.35, 0.64]**
Total events	41		226			
Heterogeneity: Chi² = 3.84, df = 1 (P = 0.05); I² = 74%						
Test for overall effect: Z = 4.90 (P < 0.00001)						
**Among untested blastocyst**						
Marcos 2015	39	94	205	408	17.8%	0.83 [0.64, 1.07]
Sciorio 2020a	97	259	539	1038	49.8%	0.72 [0.61, 0.85]
Sciorio 2020b	14	30	53	84	6.5%	0.74 [0.49, 1.12]
**Subtotal (95% CI)**		**383**		**1530**	**74.1%**	**0.75 [0.65, 0.85]**
Total events	150		797			
Heterogeneity: Chi² = 0.74, df = 2 (P = 0.69); I² = 0%						
Test for overall effect: Z = 4.26 (P < 0.0001)						
**Total (95% CI)**		**543**		**1960**	**100.0%**	**0.68 [0.60, 0.77]**
Total events	191		1023			
Heterogeneity: Chi² = 9.96, df = 4 (P = 0.04); I² = 60%						
Test for overall effect: Z = 6.24 (P < 0.00001)						
Test for subgroup differences: Chi² = 7.48, df = 1 (P = 0.006), I² = 86.6%						

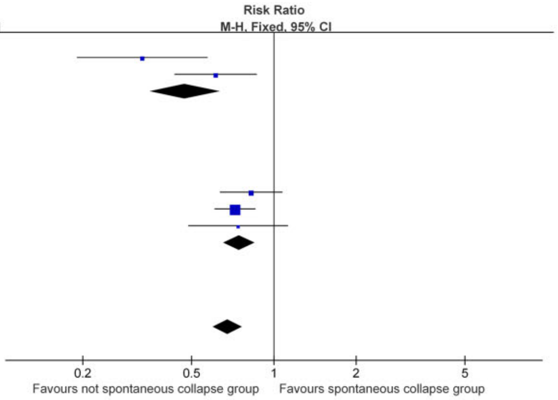

Supplementary Figure S1. **Sensitivity analysis for ongoing pregnancy rates (fixed-effect model).** Subgroup analyses for blastocysts that underwent preimplantation genetic testing (PGT) for aneuploidy or untested blastocysts were performed.
