# Supplementary material for: Spontaneous collapse as a prognostic marker for human blastocysts: a systematic review and meta-analysis
Source: Hum Reprod. 2023 Aug 15;38(10):1891–900. doi: 10.1093/humrep/dead166 (PMC10546075; doi:10.1093/humrep/dead166)
Supplement: dead166_Supplementary_Figure_S2 [file dead166_supplementary_figure_s2.pdf]

Human Reproduction, 2023, 1–1
https://doi.org/10.1093/humrep/dead166
Supplementary Data

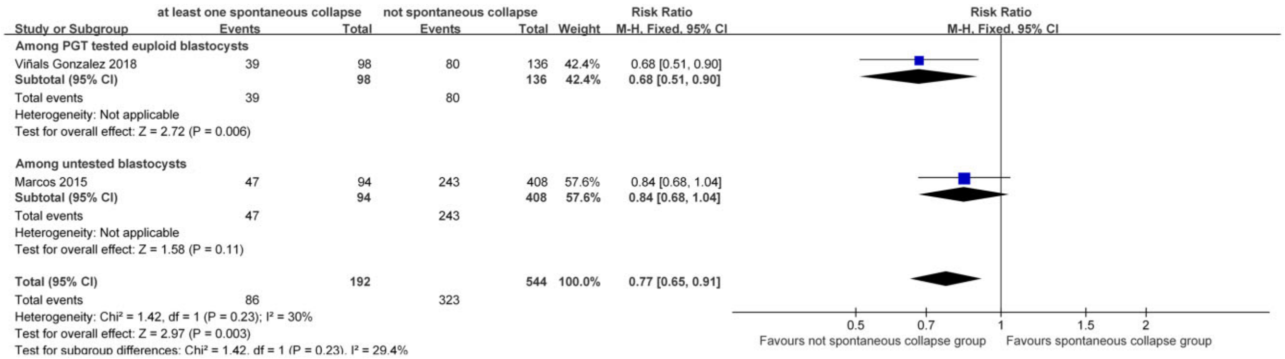

Supplementary Figure S2. Sensitivity analysis for clinical pregnancy rates (fixed-effect model). Subgroup analyses for blastocysts that underwent preimplantation genetic testing (PGT) for aneuploidy or untested blastocysts were performed.
