# Supplementary material for: Spontaneous collapse as a prognostic marker for human blastocysts: a systematic review and meta-analysis
Source: Hum Reprod. 2023 Aug 15;38(10):1891–900. doi: 10.1093/humrep/dead166 (PMC10546075; doi:10.1093/humrep/dead166)
Supplement: dead166_Supplementary_Figure_S3 [file dead166_supplementary_figure_s3.pdf]

Human Reproduction, 2023, 1–1
https://doi.org/10.1093/humrep/dead166
Supplementary Data

Study or Subgroup	at least one spontaneous collapse Events	Total	not spontaneous collapse Events	Total	Weight	Risk Ratio M-H, Fixed, 95% CI
**Among PGT tested euploid blastocysts**						
Cimadomo 2022	85	218	146	321	70.5%	0.86 [0.70, 1.05]
**Subtotal (95% CI)**		**218**		**321**	**70.5%**	**0.86 [0.70, 1.05]**
Total events	85		146			
Heterogeneity: Not applicable						
Test for overall effect: Z = 1.47 (P = 0.14)						
**Among untested blastocysts**						
Bodri 2016	28	127	54	150	29.5%	0.61 [0.41, 0.90]
**Subtotal (95% CI)**		**127**		**150**	**29.5%**	**0.61 [0.41, 0.90]**
Total events	28		54			
Heterogeneity: Not applicable						
Test for overall effect: Z = 2.46 (P = 0.01)						
**Total (95% CI)**		**345**		**471**	**100.0%**	**0.78 [0.65, 0.94]**
Total events	113		200			
Heterogeneity: Chi² = 2.26, df = 1 (P = 0.13); I² = 56%						
Test for overall effect: Z = 2.61 (P = 0.009)						
Test for subgroup differences: Chi² = 2.24, df = 1 (P = 0.13), I² = 55.3%						

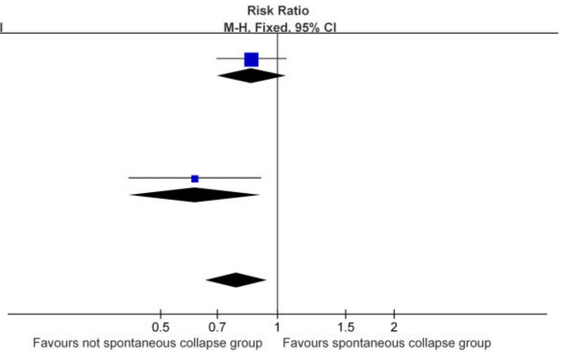

Supplementary Figure S3. **Sensitivity analysis for live birth rates (fixed-effect model).** Subgroup analyses for blastocysts that underwent preimplantation genetic testing (PGT) for aneuploidy or untested blastocysts were performed.
