# Supplementary material for: Spontaneous collapse as a prognostic marker for human blastocysts: a systematic review and meta-analysis
Source: Hum Reprod. 2023 Aug 15;38(10):1891–900. doi: 10.1093/humrep/dead166 (PMC10546075; doi:10.1093/humrep/dead166)
Supplement: dead166_Supplementary_Figure_S4 [file dead166_supplementary_figure_s4.pdf]

OXFORD
human reproduction

*Human Reproduction*, 2023, 1–1
https://doi.org/10.1093/humrep/dead166
Supplementary Data

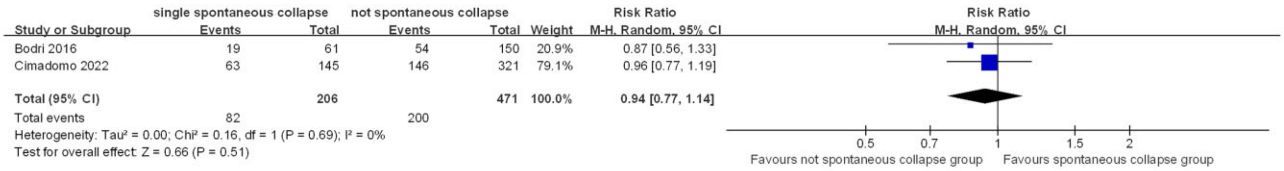


**Supplementary Figure S4.** Live birth rates between single spontaneous collapse and no spontaneous collapse.
