# Supplementary material for: Spontaneous collapse as a prognostic marker for human blastocysts: a systematic review and meta-analysis
Source: Hum Reprod. 2023 Aug 15;38(10):1891–900. doi: 10.1093/humrep/dead166 (PMC10546075; doi:10.1093/humrep/dead166)
Supplement: dead166_Supplementary_Figure_S5 [file dead166_supplementary_figure_s5.pdf]

Human Reproduction, 2023, 1–1
https://doi.org/10.1093/humrep/dead166
Supplementary Data

	multiple spontaneous collapse		not spontaneous collapse			Risk Ratio
Study or Subgroup	Events	Total	Events	Total	Weight	M-H, Random, 95% CI
Bodri 2016	9	66	54	150	38.7%	0.38 [0.20, 0.72]
Cimadomo 2022	22	73	146	321	61.3%	0.66 [0.46, 0.96]
**Total (95% CI)**		**139**		**471**	**100.0%**	**0.53 [0.31, 0.92]**
Total events	31		200			

Heterogeneity: Tau² = 0.09; Chi² = 2.24, df = 1 (P = 0.13); I² = 55%
Test for overall effect: Z = 2.28 (P = 0.02)

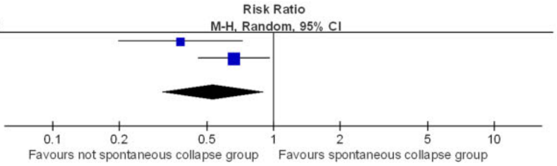

Supplementary Figure S5. Live birth rates between multiple spontaneous collapse and no spontaneous collapse.
