# Supplementary material for: Spontaneous collapse as a prognostic marker for human blastocysts: a systematic review and meta-analysis
Source: Hum Reprod. 2023 Aug 15;38(10):1891–900. doi: 10.1093/humrep/dead166 (PMC10546075; doi:10.1093/humrep/dead166)
Supplement: dead166_Supplementary_Figure_S6 [file dead166_supplementary_figure_s6.pdf]

OXFORD
human reproduction

Human Reproduction, 2023, 1–1
https://doi.org/10.1093/humrep/dead166
Supplementary Data

	single spontaneous collapse		not spontaneous collapse			Risk Ratio
Study or Subgroup	Events	Total	Events	Total	Weight	M-H, Random, 95% CI
Cimadomo 2022	207	540	486	1025	100.0%	0.81 [0.71, 0.92]
**Total (95% CI)**		**540**		**1025**	**100.0%**	**0.81 [0.71, 0.92]**
Total events	207		486			
Heterogeneity: Not applicable						
Test for overall effect: Z = 3.34 (P = 0.0008)						

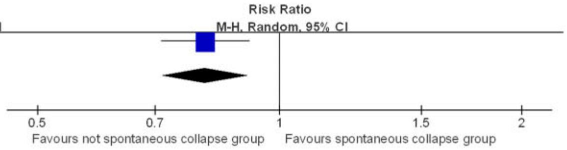

Supplementary Figure S6. Euploid embryo rates between single spontaneous collapse and no spontaneous collapse.
