# Supplementary material for: Spontaneous collapse as a prognostic marker for human blastocysts: a systematic review and meta-analysis
Source: Hum Reprod. 2023 Aug 15;38(10):1891–900. doi: 10.1093/humrep/dead166 (PMC10546075; doi:10.1093/humrep/dead166)
Supplement: dead166_Supplementary_Figure_S7 [file dead166_supplementary_figure_s7.pdf]

Human Reproduction, 2023, 1–1
https://doi.org/10.1093/humrep/dead166
Supplementary Data

	multiple spontaneous collapse		not spontaneous collapse			Risk Ratio
Study or Subgroup	Events	Total	Events	Total	Weight	M-H, Random, 95% CI
Cimadomo 2022	113	378	486	1025	100.0%	0.63 [0.53, 0.75]
**Total (95% CI)**		**378**		**1025**	**100.0%**	**0.63 [0.53, 0.75]**
Total events	113		486			
Heterogeneity: Not applicable						
Test for overall effect: Z = 5.40 (P < 0.00001)						

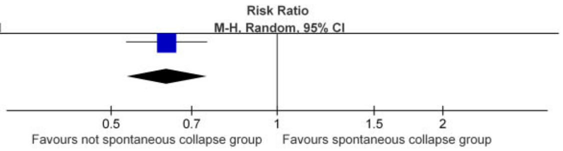

Supplementary Figure S7. Euploid embryo rates between multiple spontaneous collapse and no spontaneous collapse.
