# Supplementary material for: Spontaneous collapse as a prognostic marker for human blastocysts: a systematic review and meta-analysis
Source: Hum Reprod. 2023 Aug 15;38(10):1891–900. doi: 10.1093/humrep/dead166 (PMC10546075; doi:10.1093/humrep/dead166)
Supplement: dead166_Supplementary_Table_S1 [file dead166_supplementary_table_s1.pdf]

OXFORD

human reproduction

Human Reproduction, 2023, 1–1
https://doi.org/10.1093/humrep/dead166
Supplementary Data

Supplementary Table S1. Risk of bias assessment by the QUIPS tool.

Study ID	Study participation	Study attrition	Prognostic factor measurement	Outcome measurement	Study confounding	Statistical analysis and reporting
Bodri (2016)	Moderate risk	Moderate risk	Low risk	Low risk	Low risk	Moderate risk*
Cimadomo (2022)	Moderate risk	Moderate risk	Low risk	Low risk	Low risk	Moderate risk*
Gazzo (2020)	Moderate risk	Moderate risk	Low risk	Low risk	Moderate risk	Moderate risk
Marcos (2015)	Moderate risk	Moderate risk	Low risk	Low risk	Moderate risk	Moderate risk
Sciorio (2020a)	Moderate risk	Moderate risk	Low risk	Low risk	Low risk	Moderate risk*
Sciorio (2020b)	Moderate risk	Moderate risk	Low risk	Low risk	Moderate risk	Moderate risk
Viñals Gonzalez (2018)	Moderate risk	Moderate risk	Low risk	Low risk	Moderate risk	Moderate risk

QUIPS: Quality In Prognosis Studies; Study ID: first author and publication year.

* Although these studies considered potential confounders on critical outcomes in our meta-analysis and performed multiple regression analyses, the results could not be merged in one meta-analysis due to insufficient data (only summarized in Table 2). We used unadjusted data in the main meta-analyses and rated these studies as moderate risk on this domain.
