# Supplementary material for: Spontaneous collapse as a prognostic marker for human blastocysts: a systematic review and meta-analysis
Source: Hum Reprod. 2023 Aug 15;38(10):1891–900. doi: 10.1093/humrep/dead166 (PMC10546075; doi:10.1093/humrep/dead166)
Supplement: dead166_Supplementary_Data_File [file dead166_supplementary_data_file.pdf]

OXFORD
human reproduction

Human Reproduction, 2023, 1–1
https://doi.org/10.1093/humrep/dead166
Supplementary Data

Supplementary data file

Search strategies

All databases were searched on 10 October 2022

A. PubMed

1 "Blastocyst/abnormalities"[Mesh]
2 ((((((‘blastocyst collapse’[Title/Abstract]) OR (‘spontaneous collapse’[Title/Abstract])) OR (‘blastocyst contractions’[Title/Abstract])) OR (‘blastocyst shrinkage’[Title/Abstract])) OR (‘embryo kinetics’[Title/Abstract])) OR (‘blastocyst expansion’[Title/Abstract])) OR (‘embryonic competence’[Title/Abstract])
3 (‘Time Lapse’[Title/Abstract]) OR (‘Time Lapsed’[Title/Abstract])
4 "Time-Lapse Imaging"[Mesh]
5 1 OR 2
6 3 OR 4
7 5 AND 6
8 ("Animals"[Mesh]) NOT (("Humans"[Mesh]) AND ("Animals"[Mesh]))
9 7 NOT 8

B. Embase

1 ‘blastocyst collapse’:ti,ab OR ‘spontaneous collapse’:ti,ab OR ‘blastocyst contractions’:ti,ab OR ‘blastocyst shrinkage’:ti,ab OR ‘embryo kinetics’:ti,ab OR ‘blastocyst expansion’:ti,ab OR ‘embryonic competence’:ti,ab
2 ‘time lapse imaging’/exp OR ‘time lapse’:ti,ab OR ‘time lapsed’:ti,ab
3 1 AND 2
4 ‘animals’/exp OR ‘invertebrate’/exp OR ‘animal experiment’ OR ‘animal model’ OR ‘animal tissue’ OR ‘animal cell’ OR ‘nonhuman’
5 ‘human’ OR ‘normal human’ OR ‘human cell’
6 4 AND 5
7 4 NOT 6
8 3 NOT 7

C. Cochrane Library

1 (‘blastocyst collapse’ OR ‘spontaneous collapse’ OR ‘blastocyst contractions’ OR ‘blastocyst shrinkage’ OR ‘embryo kinetics’ OR ‘blastocyst expansion’ OR ‘embryonic competence’):ti,ab,kw
2 [mh "Time-Lapse Imaging"]
3 (‘Time Lapse’ OR ‘Time Lapsed’):ti,ab,kw
4 2 OR 3
5 1 AND 4
